# Impact of Dietary Restriction Regimens on Mitochondria, Heart, and Endothelial Function: A Brief Overview

**DOI:** 10.3389/fphys.2021.768383

**Published:** 2021-12-16

**Authors:** Cristina Elena Savencu, Adina Linţa, Gianina Farcaş, Anca Mihaela Bînă, Octavian Marius Creţu, Daniel Claudiu Maliţa, Danina Mirela Muntean, Adrian Sturza

**Affiliations:** ^1^Faculty of Dentistry, Department of Dental Prostheses Technology, Victor Babeş University of Medicine and Pharmacy, Timişoara, Romania; ^2^Faculty of Medicine, Department of Functional Sciences - Pathophysiology, Victor Babeş University of Medicine and Pharmacy, Timişoara, Romania; ^3^Faculty of Medicine, Centre for Translational Research and Systems Medicine, Victor Babeş University of Medicine and Pharmacy, Timişoara, Romania; ^4^Faculty of Medicine, Department of Surgery - Surgical Semiotics I, Victor Babeş University of Medicine and Pharmacy, Timişoara, Romania; ^5^Faculty of Medicine, Centre for Hepato-Biliary and Pancreatic Surgery, Victor Babeş University of Medicine and Pharmacy, Timişoara, Romania; ^6^Faculty of Medicine, Department of Radiology and Medical Imagistics, Victor Babeş University of Medicine and Pharmacy, Timişoara, Romania

**Keywords:** intermittent fasting, caloric restriction, cardiovascular disease, mitochondrial function, endothelial dysfunction

## Abstract

Caloric restriction (CR) and intermittent fasting (IF) are strategies aimed to promote health beneficial effects by interfering with several mechanisms responsible for cardiovascular diseases. Both dietary approaches decrease body weight, insulin resistance, blood pressure, lipids, and inflammatory status. All these favorable effects are the result of several metabolic adjustments, which have been addressed in this review, i.e., the improvement of mitochondrial biogenesis, the reduction of reactive oxygen species (ROS) production, and the improvement of cardiac and vascular function. CR and IF are able to modulate mitochondrial function *via* interference with dynamics (i.e., fusion and fission), respiration, and related oxidative stress. In the cardiovascular system, both dietary interventions are able to improve endothelium-dependent relaxation, reduce cardiac hypertrophy, and activate antiapoptotic signaling cascades. Further clinical studies are required to assess the long-term safety in the clinical setting.

## Introduction

Increased energy intake with a continuous positive, energetic balance is obviously associated with obesity and results in high cardiometabolic morbidity and mortality (Pierce et al., 2008). An increasing number of experimental and clinical studies have lately addressed the beneficial effects of reduced caloric intake. Accordingly, caloric restriction (CR) is able to prevent the development of cardiovascular disease (CVD), type 2 diabetes mellitus, cancer, and neurodegenerative disorders (Speakman and Mitchell, 2011; Di Daniele et al., 2021; Senesi et al., 2021), as well as pathologies with frequent association with each other (Ristow, 2004; Albai et al., 2020). While early research in this field was focused on the effects of conventional hypocaloric diets with continuous energy restriction, in the past decade, a new model of dietary intervention, i.e., intermittent fasting (IF), has gained scientific interest. IF is a dietary model that includes alternation of periods with reduced caloric intake with periods of unrestricted feeding. Even though both dietary models can result in a decreased energy intake, the long-term adherence to CR is more difficult as compared with IF (Wegman et al., 2015; Lee et al., 2020). The most studied models of IF used CR on 2 days/week (5:2), alternate day energy restriction (energy restriction of 60–70% below estimated requirements) and time-restricted feeding (TRF) (timely restriction of calories intake during a specific time window during the day) (Nowosad and Sujka, 2021). Regardless of the model, IR and CR have been systematically associated with several beneficial effects in animal models and humans (Di Daniele et al., 2021). The cardiometabolic benefits can be summarized as follows: decrease in body weight and fat mass, improvement of insulin sensitivity, decrease in blood pressure, lipid-lowering effect, and decrease in the level of serum inflammatory markers (Johnson et al., 2016; Brandhorst and Longo, 2019). Mechanistically, all these beneficial effects are the results of several metabolic adaptations, which will be discussed in this review: the improvement of mitochondrial biogenesis, the decreased reactive oxygen species (ROS) production, and the improvement of cardiac and vascular function.

## Mitochondrial Metabolism

Mitochondrial dysfunction has emerged in the past decade as a central pathomechanism of cardiometabolic disorders. One important trigger in the normalization of mitochondrial quality and quantity in disease is represented by calorie restriction diets. There are several studies demonstrating that CR and IF may develop the protective effects of mitochondria, but the exact mechanisms underlying the improvement in mitochondrial function and dynamics are not fully understood. We further summarized the main studies aimed at investigating the effects of both CR and IF in animal experiments and humans. The main effects of CR and IF dietary interventions on mitochondrial function, endothelial dysfunction, and cardiovascular parameters are presented in Tables 1, 2.

**Table 1:** The main effects of caloric restriction (CR) dietary intervention on mitochondrial function, endothelial dysfunction, and cardiovascular parameters.

**Reference**	**Targeted mechanism**	**Main effects**	**Type of study**
Sohal et al., 1994	Mitochondrial respiration	Reduction of mitochondrial state 4 or resting respiratory rate	Animal model (9, 16 and 23 months-old C57BL/6NNia mice, 40% CR for 1 month)
Gabbita et al., 1997	Mitochondrial respiration	Limitation of the oxy-radical production	Animal model (Brown Norway rats, 40% CR, for 47 months)
Gredilla et al., 2001	Mitochondrial respiration	Modulation of complex I; reduced oxygen radicals per unit electron flow in the respiratory chain	Animal model (8 weeks-old Wistar rats, 40% CR for 6 weeks or 1 year)
Nisoli et al., 2005	Mitochondrial respiration	Increased ATP concentrations in white adipose tissue	Animal model (8-weeks-old wild type and eNOS null mutant eNOS^−/−^ mice, alternate day fasting for 3 and 12 months)
Shinmura et al., 2011	Mitochondrial respiration	Preservation of state 3 respiration and increasing of respiratory control index in the presence of pyruvate/malate in ischemic-reperfused heart	Animal model (26-week-old Fischer 344 rats, 10% CR for 2 weeks, followed by 40% CR for 24 weeks)
Serna et al., 2020	Mitochondrial respiration	Lower maximal respiratory rates in heart and a reduced rate of hydrogen peroxide release	Animal model (12 weeks-old Sprague-Dawley rats, 2 weeks adaptation, followed by 40% CR for 6 months)
Lambert et al., 2004	Mitochondrial respiration	Increased state 4 mitochondrial respiration rate in brown adipose tissue	Animal model (60 days-old Brown Norway rats, 50% CR for 4.5 months or 17 months)
Nisoli et al., 2005	Dynamics (Fusion/Fission)	Increased expression of mitofusin (MFN) 1 and 2 proteins in animals subjected to CR	Animal model (8-week-old wild type and eNOS null mutant eNOS^−/−^ mice, alternate day fasting for 3 and 12 months)
Khraiwesh et al., 2013	Dynamics (Fusion/Fission)	Increased expression of mitochondrial fission proteins (FIS1 and DRP1) in animals subjected to CR	Animal model (10 week-old C57BL/6 mice, 40% CR for 6 months)
Colom et al., 2007	ROS production	Reduction of maximal H_2_O_2_ production of heart mitochondrial complexes I and III (values from females were lower compared to males)	Animal model (15-month-old Wistar rats, 40% CR for 3 months)
Sohal et al., 1994	ROS production	The rates of mitochondrial superoxide and hydrogen peroxide generation increased with age and are higher in the *ad libitum* than CR mice	Animal model (9, 16 and 23 months-old C57BL/6NNia mice, 40% CR for 1 month)
López-Torres et al., 2002	ROS production	Long-term CR decreased the rate of mitochondrial H_2_O_2_ generation (by 45%) and lowered oxidative damage to mtDNA (by 30%)	Animal model (12 months-old Wistar rats, 40% CR for 12 months)
Barros et al., 2004	ROS production	H_2_O_2_ release/O_2_ consumption ratios in yeasts grown in 2% glucose were significantly higher than those of CR mitochondria	Saccharomyces cerevisiae yeast cells (cultured in medium containing 0.5 or 2% glucose)
Nisoli et al., 2005	ROS production	Increased the expression of eNOS with higher concentrations of cGMP in white adipose tissue and in several other tissues	Animal model (8-week-old wild type and eNOS null mutant eNOS^−/−^ mice, alternate day fasting for 3 or 12 months)
Kobara et al., 2015	ROS production	Reduced NADPH oxidase activity and mitochondrial ROS production; Increased antioxidant systems—myocardial glutathione peroxidase and superoxide dismutase	Animal model (6 weeks-old c57BL6 mice, 40% CR for 4 weeks)
David et al., 2018	ROS production	Reduction of H_2_O_2_ generation and protein sulfhydryl oxidation in the setting of isoproterenol-induced hypertrophy	Animal model (6 weeks-old Swiss mice, 40% CR for 3 weeks)
Waldman et al., 2018	ROS production	Reduction of MDA levels (malondialdehyde) in the serum and increased the level of PGC-1α protein	Animal model (wild type or db/db mice 12–14 weeks old, 2 weeks 10% CR, followed by 2 weeks of 35% CR)
Shinmura et al., 2011	ROS production	Reduction of the maximal H_2_O_2_ production	Animal model (26 week-old Fischer 344 rats, 40% CR for 6 months)
Chen et al., 2012	ROS production	Reduction of H_2_O_2_ production from Complex I and III	Animal model (14 weeks old C57BL/6 mice, 40% CR for 1 month)
Judge et al., 2004	ROS production	Increase the activity of mitochondrial superoxide dismutase (SOD) and glutathione peroxidase (GPX) activities	Animal model (14 weeks old male Fischer 344 rats, 1 week 10% CR, 1 week 25% CR and 40% CR for 2 months)
Colom et al., 2007	ROS production	Decreased heart mitochondrial H_2_O_2_ generation	Animal model (15-month-old male and female Wistar rats, 40% CR for 3 months)
Niemann et al., 2010	ROS production	Improvement of mitochondrial function with lower oxidative damage.	Animal model (6 and 24 month-old Sprague–Dawley rats, 40% CR for 6 months)
Qiu et al., 2010	ROS production	Protective effects of CR on oxidative stress are diminished in mice lacking SIRT3	Animal model (6 months-old SIRT^−/−^ mice, 30% CR for 6 months)
Facchini et al., 2003	Blood pressure, lipid profile, HOMA index	CR decreased blood pressure, total cholesterol and LDL-cholesterol and the 10-yr risk of CVD-disease was reduced by 30%; homeostasis model assessment of insulin resistance (HOMA-IR) decreased during weight loss in the CR group.	Human study (3 weeks of CR (reduction with 500 kcal/day from basal energy expenditure) and high-intensity exercise training)
Waldman et al., 2018	Blood pressure, lipid profile, cardiac fibrosis and hypertrophy	CR attenuated the angiotensin 2-induced hypertension in diabetic mice, improved lipid, reduced cardiac fibrosis and leukocyte infiltration, decreased genes related to cardiac remodeling	Animal model (12–14 weeks old wild type or db/db mice, 2 weeks 10% CR, followed by 35% CR for 2 weeks)
Deus et al., 2019	Heart contractility	Severe CR resulted in a 50% decrease in body weight, impaired SERCA2a activity and heart contractility	Animal model (60-day-old Wistar-Kyoto rats, 50% CR for 90 days)
Finckenberg et al., 2012	Cardiac fibrosis and hypertrophy	Reduction of angiotensin II-induced cardiomyocyte hypertrophy, vascular inflammation, cardiac damage and fibrosis, cardiomyocyte apoptosis, and cardiac atrial natriuretic peptide mRNA overexpression	Animal model (4-week-old double transgenic rats and age-matched normotensive Sprague-Dawley rats, 40% CR for 1 month)
Palee et al., 2020	Body weight, lipid profile, blood pressure	Decrease in body weight and visceral fat deposition, decreased of plasma cholesterol levels, increased insulin sensitivity and decrease of BP	Animal model (6 months of 40% CR in high-fat-diet obese rats)
Fontana et al., 2004	Lipid profile, blood glucose, blood pressure	Improvement of lipid profile, fasting glucose and insulin, normalization of BP and lower carotid artery IMT	Human study (individuals who have been practicing CR for periods from 3 to 15 years)
Niemann et al., 2010	Cardiac hypertrophy	Reduction of cardiomyocyte hypertrophy and of natriuretic peptides BNP/ANP, of pro-apoptotic Bcl-xS/Bcl-xL	Animal model (6 months-old Sprague–Dawley rats, 40% CR for 6 months)
Almeida et al., 2020	Vascular relaxation (contractility and endothelial dependent relaxation)	Severe CR in rats (for 2 weeks) was responsible for endothelial dysfunction in mesenteric arteries and for ischemia–reperfusion-induced arrhythmias and cardiac pathology	Animal model (4 months-old Fischer rats, 60% CR for 2 weeks)
Zanetti et al., 2010	Vascular relaxation (endothelial dependent relaxation) and NOS isoforms level	Improvement of endothelium-dependent vasorelaxation, increase in eNOS level and decrease of iNOS (study in aged rats)	Animal model (6 and 24 months-old Fischer 344 rats, 26% CR for 3 weeks)
Ketonen et al., 2010	Vascular relaxation and ROS production	Reverse of high-fat diet-induced endothelial dysfunction and vascular superoxide production in C57Bl/6 mice	Animal model (3–4 weeks-old C57Bl/6 mice with induced obesity—high fat diet for 150 days, followed by 30% CR for 50 days)
Sasaki et al., 2002	Vascular relaxation (evaluated by brachial artery flow mediated dilation)	Improvement of endothelium-dependent vasodilation in obese patients with essential hypertension	Human study (obese patients with essential hypertension before and after 2 weeks on a low-calorie diet–800 kcal/day)
Rippe et al., 2010	Carotid artery endothelium-dependent dilation, ROS production	Short-time CR reverse of vascular endothelial dysfunction in old mice by increasing nitric oxide and reducing oxidative stress (decrease of NADPH oxidase-related ROS production)	Animal model (young–5–8 months, and older–28–30 months, B6D2F1 mice, 30% CR for 8 weeks)
Donato et al., 2013	Pulse wave velocity (PWV, arterial stiffness), carotid artery wall thickness, endothelium-dependent relaxation, ROS production	Long CR reduces oxidative stress and preserves nitric oxide bioavailability and function in arteries of aged mice	Animal model (young–5–7 months, and older–30–31 months, B6D2F1 mice, 40% CR life-long)

**Table 2:** The main effects of intermittent fasting (IF) dietary intervention on mitochondrial function, endothelial dysfunction, and cardiovascular parameters.

**Reference**	**Targeted mechanism**	**Main effects**	**Type of study**
Real-Hohn et al., 2018	Mitochondrial respiration	Increased O_2_ flux rate related to ATP production and greatest RCR values	Animal model (60 day-old Wistar rats, IF together with high intensity intermittent exercise for 8 weeks)
Castello et al., 2010	ROS production	Reduced oxidative stress (improved the level of glutathione) and to decreased inflammatory status in the heart during aging	Animal model (2 months-old Sprague Dawley rats, alternate-day fasting until 6, 12, or 24 months)
Real-Hohn et al., 2018	ROS production	a strong reduction in MDA levels in the IF/HIIE group; the IF/HIIE group presented lower levels of plasma protein oxidation.	Animal model (60 day-old Wistar rats, IF together with high intensity intermittent exercise for 8 weeks)
Ahmet et al., 2005	Infarct size, cardiac remodeling	Reduction of the infarct size, cardiomyocyte apoptosis and infiltration with neutrophils and macrophages in rats subjected for experimental myocardial infarction; improvement of left ventricular (LV) remodeling and diastolic posterior wall thickness	Animal model (2 month-old Sprague-Dawley rats, alternate day fasting—every other day, 3 months)
Katare et al., 2009	Infarct size, cardiac remodeling	Improvement of the rate survival after large myocardial infarction; activation of PI3kinase/Akt and VEGF pathway	Animal model (8-10 weeks-old Wistar rats; experimental myocardial infarction; after 2 week initiation of alternate day feeding until day 100 from onset of myocardial infarction)
Castello et al., 2010	Interference with cytokine activity	Reduced the amount of TNFα, IL6, and IL1β in aged myocardium animals together with reduction of TGF-β1, collagen contents and NF-κB DNA binding activity	Animal model (2 months-old Sprague Dawley rats, alternate day fasting—every other day, for 6, 12, or 24 months)
Okoshi et al., 2019	Cardiac remodeling	Reduction in total mortality after myocardial infarction; reduction of the left ventricular (LV) diastolic posterior wall thickness	Animal model (2 months-old Wistar rats, alternate day fasting—every other day, for 12 weeks, and subjected for myocardial infarction)
Basilio et al., 2020	Glycemic control and cardiac remodeling, apoptosis	Improved glycemic values; reduction of cardiac interstitial collagen fraction; a low expression of proapoptotic gene BAX and of cytochrome C, and increased expression of the antiapoptotic protein Bcl-2	Animal model (60 days-old Wistar rats, alternate day fasting—every other day, and exercise training, for 12 weeks)
Varady et al., 2009	Lipid profile, cardiovascular risk	Improved lipid profile in obese patients	Human study [10-week trial in obese subjects, 3 phases: (1) a 2-week control phase, (2) a 4-week alternate day fasting controlled food intake phase, and (3) a 4-week alternate day fasting self-selected food intake phase]
Carvalho et al., 2021	Cardiac remodeling, apoptosis	Reduction of collagen interstitial fraction, expression of proapoptotic gene BAX and of cytochrome C and increased expression of the antiapoptotic protein Bcl-2.	Animal model (60 day-old Wistar rats, alternate day fasting together with high intensity training for 12 weeks)
Hoddy et al., 2016	Insulin-resistance and endothelial function	IF (alternate-day-fasting) reduce HOMA-IR in patients who are severely insulin resistant; no effects on endothelial function.	Human study (obese non-diabetic subjects, 8-week of alternate day fasting–25% energy intake “fast day”, alternated with *ad libitum* intake “feast day”)
Bhutani et al., 2013	Vascular relaxation (evaluated by brachial artery flow mediated dilation)	IF (alternate-day-fasting)—an effective intervention to improve vascular endothelial function (improved brachial artery flow mediated dilation in obese subjects)	Human study (obese subjects, 12 weeks of alternate day fasting—*ad libitum* “feed day” alternated with a 75% energy restriction “fast day,” combined with endurance exercise)
Razzak et al., 2011	Endothelium dependent relaxation	IF reduces weight of male rats and improves their aortic endothelium-dependent vasorelaxation	Animal model (Wistar rats, alternate day fasting for 2 months)
Headland et al., 2018	Vascular relaxation (evaluated by brachial artery flow mediated dilation)	Two consecutive days of energy restriction (IF–5:2 diet) has no effect on endothelial function	Human study (4 weeks of low energy diet–500 calories for women, 600 calories for men, on two consecutive days per week and 5 days of habitual eating)

### Effects of CR and IF on Mitochondrial Dynamics

#### Caloric Restriction

Mitochondria are dynamic intracellular organelles that fuse and divide, being actively recruited to different cellular locations. Three large GTPases are required in the process of mitochondrial fusion: protein OPA1 (Mitochondrial Dynamin-Like GTPase) from the inner mitochondrial membrane, and protein mitofusins 1 and 2 (MFN1 and MFN2) at the outer membrane. Importantly, the expression of MFN1 and MFN2 was observed to be increased (in the white and brown adipose tissue, heart, brain, and liver) in animals subjected to CR (3 or 12 months) (Nisoli et al., 2005). This effect was mediated by endothelial nitric oxide synthase (eNOS) since it was absent in eNOS KO animals (Nisoli et al., 2005).

Mitochondrial fission requires GTPase dynamin-related protein 1 (DRP1) and mitochondrial fission protein 1 (FIS1) from the outer membrane. Khraiwesh et al. analyzed the ultrastructural changes and markers of fission/fusion in hepatocyte mitochondria from mice submitted to 40% CR for a period of 6 months. The results showed that proteins related to mitochondrial fission (FIS1 and DRP1) increased with CR, but the three fusion proteins examined (MFN1, MFN2, and OPA1) did not exhibit changes (Khraiwesh et al., 2013).

Mitochondrial fusion and fission are crucial for mitochondrial function. In an experimental model aimed to characterize the impact of CR on mitochondrial morphology and dynamics in muscular tissue, it was reported that CR (starting with a 20% restriction for 2 weeks, followed by a 40% CR for 13 months) attenuated the age-related mitochondrial fragmentation and also increased the muscle level of fusion protein MFN2 (Faitg et al., 2019).

At present, the effects of IF on mitochondrial ultrastructure and dynamics have been less explored.

### Effects of CR and IF on Mitochondria-Related Oxidative Stress

#### Caloric Restriction

Mitochondria are the major sources of intracellular ROS being also vulnerable to oxidative stress. There are numerous studies in animals and humans showing the beneficial effects of diet intervention on mitochondria-related ROS production. Importantly, a decrease in ROS generation has been associated with caloric intake and age, as reported by a pioneering study where the level of mitochondrial superoxide and hydrogen peroxide was significantly higher in mice fed *ad libitum* vs. mice subjected to 40% CR for a period of 23 months (Sohal et al., 1994). The long-term exposure to CR is able to reduce not only the oxidative stress but also the oxidative damage of the mitochondrial DNA (by 30%) (López-Torres et al., 2002). Mechanistically, it seems that CR can influence both ROS generation and antioxidant capacity. Specifically, CR reduced the activity of NADPH oxidase and increased the activity of the antioxidant enzymes such as glutathione peroxidase (GPX) and superoxide dismutase (SOD), in the setting of cardiac hypertrophy associated with chronic pressure overload (Kobara et al., 2015). Even short periods of CR (3 weeks) were able to prevent the development of isoproterenol-induced cardiac hypertrophy via the reduction of ROS generation (decreased H_2_O_2_ and lower protein sulfhydryl oxidation) and the preservation of the antioxidant enzymes (i.e., catalase, SOD, and GPX) activity, respectively (David et al., 2018). Interestingly, severe CR in rats (complete restriction for 72 h) decreased the expression of monoamine oxidase (MAO), which is a mitochondrial enzyme at the outer mitochondrial membrane in the white adipose tissue (Iffiú-Soltész et al., 2009). MAO expression was increased in obese patients with the inflammatory status being responsible for high ROS production (Sturza et al., 2019). Apparently, CR is able to interfere with the monoaminergic system by increasing the level of noradrenaline in the brain and improving the peripheral levels of insulin, leptin, calcium, and HDLc in old animals (Portero-Tresserra et al., 2020).

Also, in an experimental model of ischemia-reperfusion aimed to investigate the effect of CR on mitochondrial function and subsequent molecular mechanisms of CR-induced cardioprotection, a significant reduction of mitochondrial oxidative stress (H_2_O_2_) in cardiac samples isolated from the CR group (6 months) (Shinmura et al., 2011). Also, in the same study, a reduction in the acetylated forms of NADH-ubiquinone oxidoreductase, i.e., the largest subunit of complex I (NDUFS1) and cytochrome bc1 has been reported in the CR group (Shinmura et al., 2011). In almost all animal studies aimed to investigate the effect of CR on oxidative stress in the heart, H_2_O_2_ was found to be reduced together with an increase in the antioxidant capacity in response to CR and with an increase in mitochondrial differentiation (mitochondrial protein/gram of tissue and mitochondrial protein/mitochondrial DNA) (Colom et al., 2007). The effect is mainly mediated by the regulation of mitochondrial complexes I and III (Colom et al., 2007), and in some experiments, the effect was present even after 1 month of CR (Chen et al., 2012). In leptin-resistant-obese mice, CR (1 month) reduced the levels of malondialdehyde (MDA) in the serum and increased the level of peroxisome proliferator-activated receptor-gamma coactivator 1 alpha (PGC-1α protein), which is a member of a family of transcription coactivators with a central role in the regulation of energy metabolism) (Waldman et al., 2018). Of note, the maximal H_2_O_2_ production from rat heart mitochondrial complexes I and III was lower in females compared to males and was decreased by a 3-month period of CR (Colom et al., 2007).

An interesting observation is the effect of CR on the antioxidant mitochondrial enzymes vs. the cytosolic ones. It was noticed that a short period of CR (2 months) in rats significantly increased the activity of mitochondrial (but not of cytosolic) SOD and GPX activity (Judge et al., 2004). Also, long periods of CR (6 months) in senescent rats alleviated mitochondrial dysfunction resulting in less oxidative damage and reduced mortality (Niemann et al., 2010).

The protective effect induced by 6 months of CR on mitochondria also involved the regulation of SIRT3 (i.e., NAD-dependent deacetylase sirtuin-3), a mitochondrial deacetylase (Qiu et al., 2010; Shinmura et al., 2011). SIRT3 deacetylase increased mitochondrial respiration and reduced ROS production *via* the activation of SOD2. In mice lacking SIRT3, the protective effects of CR were significantly reduced (Qiu et al., 2010).

#### Intermittent Fasting

The amount of studies investigating the effect of IF on mitochondrial ROS production is rather limited. A protocol of alternate-day fasting (ADF, 24 months) in rats was able to reduce oxidative stress (assessed by the increased level of glutathione) and decrease inflammatory status in the heart during aging similar to young animals (Castello et al., 2010). Also, every-other-day IF in combination with high-intensity intermittent exercise protocol optimized the metabolic pathways, with a strong reduction in MDA levels and a decrease in plasma protein oxidation (Real-Hohn et al., 2018).

### Effects of CR and IF on Mitochondrial Respiration

#### Caloric Restriction

Mitochondrial respiration is the most important mechanism of cellular energy generation, and its impairment had been linked to a plethora of pathological conditions. Several studies have investigated whether oxidative damage and impairment of mitochondrial respiration can be modulated by the reduction in caloric intake. In an experimental study in rats using a protocol of CR (40%, 23 months), it was noticed that mitochondrial state 4 increased with age (with subsequent high ROS production) in the *ad libitum* fed, but not in the CR group (Sohal et al., 1994). Importantly, CR can generate rapid effects (1 month) on the electron transport system (Chen et al., 2012). Another study was aimed to assess the impact of CR on mitochondrial respiration in the aging brain (25-month-old animals) and showed that CR was able to reduce the age-related membrane rigidization and limit the oxy-radical production, following the metabolic stimulation of mitochondria with succinate (Gabbita et al., 1997). At variance, in a study focused on the antiaging effect of CR on mitochondria isolated from different rat tissues (brown adipose tissue, liver, heart, kidney, and brain), no significant effects of CR on state 4 mitochondrial respiration rate were found in all tissues, except for the brown adipose tissue, where state 4 was ~3-fold higher as compared to mitochondria from fully fed controls (Lambert et al., 2004).

In other studies, CR (short period: 6 weeks and long period: 1 year) decreased the amount of ROS generated at the level of the respiratory chain (Gredilla et al., 2001). In mice subjected to CR diets (3 and 12 months), oxygen consumption was higher, especially in the white adipose tissue; this process was correlated with a higher ATP level (Nisoli et al., 2005). In rat hearts subjected to an ischemia-reperfusion injury episode, prior 6 months of CR preserved state 3 respiratory rate and increased the respiratory control index when pyruvate and malate were used as respiratory substrates, indicating that mitochondria isolated at the post-ischemic reperfusion were well-coupled (Shinmura et al., 2011). In a recent elegant study, the Kowalkowski group highlighted the tissue-specific changes in bioenergetics, mitochondrial calcium handling, and redox balance in cardiac and skeletal muscle mitochondria in response to CR (6 months). Accordingly, CR decreased respiration in the presence of ATP synthesis in the heart and soleus muscle. In the heart (but not in skeletal muscle), lower maximal respiratory rates and a reduced rate of hydrogen peroxide release were reported. In this model, no changes in the respiratory control ratios were found (Serna et al., 2020).

#### Intermittent Fasting

The evaluation of mitochondrial respiration in rats subjected to IF for 8 weeks showed that an every-other-day protocol lead to an improved O_2_ flux and ATP production coupling demonstrated by high respiratory control ratios. These beneficial effects were synergic when combined with a high-intensity intermittent exercise protocol (Real-Hohn et al., 2018).

## Effects of Dietary Interventions on Cardiovascular Metabolic Parameters

Despite the fact that CVD remains the leading cause of morbidity and mortality all over the world, many of the CVD risk factors can be partially controlled (and reversed) by dietary intervention and physical exercise. Currently, several studies demonstrated that CR and IF represent powerful protective mechanisms in both heart and vasculature.

### Caloric Restriction

In the heart, CR can attenuate age-related changes that occurred in the myocardium, i.e., hypertrophy, fibrosis, and apoptosis (Weiss and Fontana, 2011), and can preserve or improve diastolic function. Similarly, short periods of CR (2–3 weeks) prevented the development of cardiac hypertrophy (Kobara et al., 2015; David et al., 2018); long periods (3 months) were able to improve cardiac insulin sensitivity and to prevent the reduction in myocardial contractility associated with aging (Granado et al., 2019).

Most authors aimed to investigate whether CR can reduce risk factors for CVD and insulin resistance in non-obese humans; these authors showed that CR decreased visceral and subcutaneous abdominal adipose tissue, blood pressure, and homeostatic model assessment (HOMA) index and also improved lipid profile, and the 10-year risk of CVD was reduced by 30% (Most et al., 2018).

Importantly, the high generation of angiotensin 2 (Gavras and Gavras, 2002) and the high level of plasma lipoproteins associated with elevated triglyceride levels (Balint et al., 2011) are able to increase the cardiovascular risk and complications. CR was able to attenuate the angiotensin 2-induced hypertension in diabetic mice (Waldman et al., 2018), ameliorate angiotensin 2-induced cardiomyocyte hypertrophy, fibrosis, and apoptosis (Finckenberg et al., 2012) and to improve the lipid profile (Waldman et al., 2018). CR leads to the downregulation of the genes associated with cardiac overload, i.e., brain natriuretic peptide (Niemann et al., 2010; Waldman et al., 2018) and atrial natriuretic peptide (Niemann et al., 2010; Finckenberg et al., 2012), of genes related to remodeling (transforming growth factor-beta (TGF-β), tumor necrosis factor-alpha (TNF-α), and matrix metallopeptidase 2 (MMP2) (Waldman et al., 2018); furthermore, CR also reduced the expression of proapoptotic genes Bcl-xS/Bcl-xL, mitochondrial translocation of BAX, release of cytochrome c into cytosol, and caspase-9 activation (Niemann et al., 2010).

The CR for 3 or 12 months increased the expression of eNOS and cGMP formation in various murine tissues. This influenced mitochondrial biogenesis with increased oxygen consumption and ATP production (Nisoli et al., 2005).

Fontana et al. followed 18 individuals who had been on the CR diet for an average of 6 years and 18 age-matched healthy individuals on typical American diets. They found significantly better lipid profiles and blood pressure values in the CR group. Importantly, carotid artery IMT was 40% less in the CR group (Fontana et al., 2004). However, not only the long periods but also the short periods of CR can be beneficial. In obese patients, a short period of CR and a high-intensity exercise program (3 weeks) reduced body mass index (BMI), heart rate, and blood pressure and also improved maximal oxygen consumption during exercise (Facchini et al., 2003). In other studies in humans, a hypocaloric diet was able to reduce arterial stiffness in obese adults (Dengo et al., 2010). In the animal model of obese pre-diabetic rats, 13 weeks of CR significantly decreased body weight, visceral fat deposition, blood pressure (systolic and diastolic), and improved insulin sensitivity (HOMA index) and left ventricular function (Palee et al., 2020). Also, calcium homeostasis and cardiac mitochondrial function were normalized (Palee et al., 2020). Even short periods of CR have visible effects; thus, only 4 days with CR (reduced dietary intake by 50%) significantly reduced the blood pressure in spontaneously hypertensive rats (Young et al., 1978).

However, it has to be mentioned that severe CR impaired the SERCA2a activity and lowered the expression of L-type Ca^2+^ channels with a negative impact on the cardiac contractile function (Deus et al., 2019).

Regarding the effect of CR on endothelial dysfunction, several studies showed an improvement of the endothelium-dependent vasorelaxation in different experimental settings and by several mechanisms: increase in eNOS level and decrease of iNOS (Zanetti et al., 2010), reverse of high-fat diet-induced endothelial dysfunction and vascular superoxide production in C57Bl/6 mice (Ketonen et al., 2010), improvement of endothelium-dependent vasodilation (evaluated by brachial artery flow-mediated dilation) in obese patients with essential hypertension (Sasaki et al., 2002), reversal of vascular endothelial dysfunction in old mice by increasing nitric oxide and decreasing NADPH oxidase-related ROS production (Rippe et al., 2010), improvement of pulse wave velocity, carotid artery wall thickness, and endothelium-dependent relaxation also in aged mice (Donato et al., 2013).

However, a short period (2 weeks) of severe food restriction in rats was responsible for endothelial dysfunction in mesenteric arteries and for ischemia-reperfusion-induced arrhythmias and cardiac pathology (Almeida et al., 2020).

### Intermittent Fasting

The impact of IF diet protocols on heart metabolism was also studied. It was revealed that alternate-day-fasting can reduce left ventricular fibrosis in the aged rat hearts (Castello et al., 2010), infarct size (Ahmet et al., 2005; Okoshi et al., 2019), cardiomyocyte apoptosis, and infiltration with neutrophils and macrophages in rats subjected to myocardial infarction (Ahmet et al., 2005). Importantly, the protective effects of IF were persistent since echocardiography revealed that after 10–12 weeks after myocardial infarction, the left ventricular remodeling and diastolic posterior wall thickness was decreased in the IF group (IF regimen continued) (Ahmet et al., 2005; Okoshi et al., 2019). Of note, in these experiments, the mortality rate in the IF group was significantly reduced than in controls (Ahmet et al., 2005; Okoshi et al., 2019). Mechanistically, it was demonstrated that the activation of PI3kinase/Akt and VEGF pathway is responsible for the IF-induced protection in chronic myocardial ischemia (Katare et al., 2009). Molecular studies showed the increase of several angiogenic factors (sHIF-1-α, BDNF, and VEGF) in the fasted hearts (Katare et al., 2009). Also, it was showed an increased capillary density in the ischemic myocardium and the synthesis of VEGF by cardiomyocytes and the upregulation of antiapoptotic factors expression (Akt and Bcl-2) (Katare et al., 2009). IF activated cell survival cascade and also improved the survival rate after large myocardial infarction (Katare et al., 2009). The cardioprotective effects induced by IF are mediated by interaction with pro-inflammatory cytokines; alternate-day-fasting reduced the amount of TNFα, IL-6, and IL-1β in aged myocardium animals together with the reduction of TGF-β1, collagen contents, and NF-κB DNA-binding activity (Castello et al., 2010). Other studies aimed to investigate the influence of IF (associated with exercise training) on cardiac remodeling, which reported a reduction in cardiac interstitial collagen fraction (Basilio et al., 2020; Carvalho et al., 2021), a better glycemic control, a low expression of proapoptotic gene BAX and of cytochrome c, and increased expression of the antiapoptotic protein Bcl-2 (Carvalho et al., 2021).

Despite the positive findings in animal research, currently, there are no long-term safety data with IF diets, especially in the normal-weight population. Also, it has to be emphasized that severe/drastic IF diets (reduction with 70% of caloric intake) have been associated with adverse effects such as fatigue, irritability, mood disorders, concentration difficulties, and uncontrolled hyperphagia on the days of unrestricted feeding. Nevertheless, this is not the case for overweight and obese individuals, which followed intermittent CR diets (Katsarou et al., 2021).

Regarding the endothelial function, most of the studies using IF regimens showed beneficial effects: improved brachial artery flow-mediated dilation (Bhutani et al., 2013), improved endothelial- and non-endothelial-dependent vasodilation response and decreased blood pressure in human subjects (Ezad and Borne, 2016), and improved endothelial function in adults with metabolic syndrome after 8 weeks of IF (Guo et al., 2021). Also, there are studies with no significant effect of IF on endothelial function; two consecutive days of energy restriction (IF = 5:2 diet) had no effect on endothelial function (evaluated by brachial artery flow-mediated dilation) (Headland et al., 2018). Also, no effect of TRF on vascular endothelial function in middle-aged and older adults (Martens et al., 2020), despite other benefits (e.g., reduction of insulin resistance) (Hoddy et al., 2016) were reported. Still, TRF is an IF dietary approach used for weight loss and overall health, being able to improve metabolic syndrome parameters and cardiovascular risk in obese women (Schroder et al., 2021).

**Figure 1:**
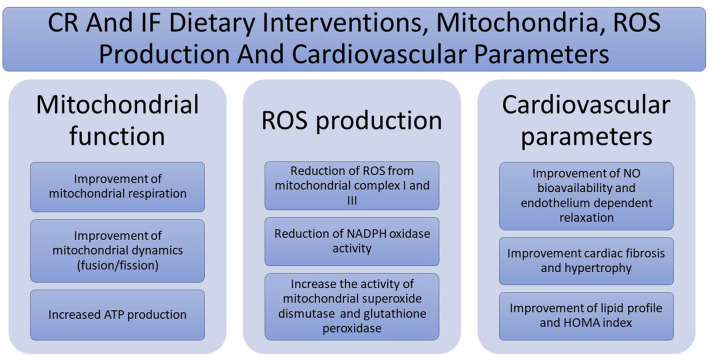
Main beneficial effects of caloric restriction (CR) and intermittent fasting (IF) dietary interventions on mitochondria, reactive oxygen species (ROS) production, and cardiovascular parameters.

Main beneficial effects of CR and IF dietary interventions on mitochondria, ROS production, and cardiovascular parameters are depicted in Figure 1.

## Conclusion

In the last years, the interest in dietary strategies aimed at decreasing weight and also counteracting CV disease is increasing. The use of CR diets, more recent of IF regimens, represents the nutritional approaches that may normalize mitochondrial, endothelial/vascular function, and cardiomyocyte/heart metabolism mechanisms that have been unequivocally proven in various experimental settings. However, large clinical trials are required to allow definitive conclusions regarding the long-term safety of these simple approaches in both prevention and therapy of cardiometabolic diseases.

## Author Contributions

DCM, DMM, and AS proposed the conception. CES and AL contributed equally to the original draft preparation. AMB, GF, and OMC were involved in all aspects of writing, editing, and decision to publish. All authors contributed to the article and approved the submitted version.

## Conflict of Interest

The authors declare that the research was conducted in the absence of any commercial or financial relationships that could be construed as a potential conflict of interest.

## Publisher's Note

All claims expressed in this article are solely those of the authors and do not necessarily represent those of their affiliated organizations, or those of the publisher, the editors and the reviewers. Any product that may be evaluated in this article, or claim that may be made by its manufacturer, is not guaranteed or endorsed by the publisher.
